# Addressing Pain for a Proper Rehabilitation Process in Patients With Severe Disorders of Consciousness

**DOI:** 10.3389/fphar.2021.628980

**Published:** 2021-02-17

**Authors:** F. Riganello, A. Soddu, P. Tonin

**Affiliations:** ^1^ Research in Advanced NeuroRehabilitation, Istituto Sant’Anna, Crotone, Italy; ^2^ Department of Physics and Astronomy, Brain and Mind Institute, Western University, London, ON, Canada; ^3^ Research in Advanced NeuroRehabilitation, Istituto Sant’Anna, Crotone, Italy

**Keywords:** disorders of consciousness, pain, rehabilitation, assessment, pharmacotherapeutic approaches

## Abstract

Consciousness constitutes a fundamental prerequisite in the individual appraisal and experience of pain. In the same way, a person needs to be able to report on pain perception. Patients who suffered a severe brain injury with disorders of consciousness (DOC) represent a spectrum of pathologies affecting patients' capacity to interact with the external world. In these patients, the most relevant aspects in response to pain are physiologic and behavioral. The treatments and management of pain are challenging issues in these patients, arising serious ethical concerns and bringing emotional load among medical staff, caregivers, and relatives. In this review, we report the importance of having a correct pain management in DOC patients, to individuate the best pharmacological treatment that can make the difference in detecting a behavioral response, indicative of a change in the level of consciousness, and in planning a more effective rehabilitative approach.

## Introduction

In 1979, the IASP approved the following definition of pain: *"An unpleasant sensory and emotional experience associated with actual or potential tissue damage, or described in terms of such damage"* coupling the sensory and emotional dimensions of the experience, as well as the association between tissue injury and pain (IASP, 1979).

The emotional experience can be described by a complex system of interacting processes characterized by affective (i.e., subjective experienced feeling), expressive (e.g., mimics, behaviors), cognitive (e.g., thoughts), and physiological (e.g., heart rate) components (Scherer et al., 2001).

In 1999 McCaffrey and Pasero reported a similar definition: "Pain is *whatever the experiencing person says it is, existing whenever* the experiencing person *says it does*" denoting the subjectivity of the pain experience (McCaffrey and Pasero, 1999, p 63). Such definition implies not only that pain may be detected when a patient reports its manifestation but that consciousness constitutes a fundamental prerequisite in the individual appraisal and experience of pain. In 2007, at the Kyoto annual meeting, the publication of the modification of the IASP Basic Pain Terminology (Loeser and Treede, 2008) was approved, with the introduction of the terms nociceptive neuron, nociception, nociception stimulus, nociceptive pain, sensitization, peripheral and central sensitization (Table 1). Independently from a more accurate terminology, a key aspect of pain remains the subjective experience and the necessity to report on it (“…*the experiencing person says it is … ”*). The importance of reporting on the pain sensation is described in a study by Clarke and colleagues on the chronic pain in older adults, recommending the narrative approach to describe and discuss the experience of pain. If this approach could represent an useful tool to assess pain in subjects who are able to refer on it (Clarke et al., 2012), it highlights the issues in assessing pain in non-communicative patients.

**TABLE 1:** International association for the study of the pain–terminology.

Pain: An unpleasant sensory and emotional experience associated with, or resembling that associated with, actual or potential tissue damage.• Pain and nociception are different phenomena. Pain cannot be inferred solely from activity in sensory neurons• Pain is always a personal experience that is influenced to varying degrees by biological, psychological, and social factors• Verbal description is only one of several behaviors to express pain; inability to communicate does not negate the possibility that a human or a nonhuman animal experiences pain			
			
Nociception	The neural process of encoding noxious stimuli	Pain threshold	The minimum intensity of a stimulus that is perceived as painful
Nociceptive neuron	A central or peripheral neuron of the somatosensory nervous system that is capable of encoding noxious stimuli	Pain tolerance level	The maximum intensity of a pain-producing stimulus that a subject is willing to accept in a given situation
Nociceptive pain	Pain that arises from actual or threatened damage to non-neural tissue and is due to the activation of nociceptors	Paresthesia	An abnormal sensation, whether spontaneous or evoked
Nociceptive stimulus	An actually or potentially tissue-damaging event transduced and encoded by nociceptors	Sensitization	Increased responsiveness of nociceptive neurons to their normal input, and/or recruitment of a response to normally subthreshold inputs
Nociceptor	A high-threshold sensory receptor of the peripheral somatosensory nervous system that is capable of transducing and encoding noxious stimuli	Central sensitization	Increased responsiveness of nociceptive neurons in the central nervous system to their normal or subthreshold afferent input
Noxious stimulus	A stimulus that is damaging or threatens damage to normal tissues	Peripheral sensitization	Increased responsiveness and reduced threshold of nociceptive neurons in the periphery to the stimulation of their receptive fields
		Central neuropathic pain	Pain caused by a lesion or disease of the central somatosensory nervous system
		Peripheral neuropathic pain	Pain caused by a lesion or disease of the peripheral somatosensory nervous system
		Nociplastic pain	Pain that arises from altered nociception despite no clear evidence of actual or threatened tissue damage causing the activation of peripheral nociceptors or evidence for disease or lesion of the somatosensory system causing the pain


Pain assessment in non-communicative patients.

### Nociception Versus Pain

In the assessment of non-communicative patients, it is essential to discriminate a reflex from higher-order behavioral responses.

Noxious stimulation implies a response of the Autonomic Nervous System (ANS). Typical physiological responses are observable in the cardiovascular reactivity, respiration, skin conductance and pupil dilatation (Kyle and McNeil, 2014; Mischkowski et al., 2018).

The nociception (i.e., the neural process of encoding noxious stimuli) refers to the perception (conscious or not) of nociceptive stimuli (an actually or potentially tissue-damaging event transduced and encoded by nociceptors) (Loeser and Treede, 2008), eliciting the activation of an extensive cortical network (i.e. somatosensory, insular, and cingulate areas, as well as frontal and parietal areas) (Coghill et al., 2003; Chatelle et al., 2014). The transmission of the information of the nociceptive stimulation follows the *via* spinothalamic tract to reach the thalamus and the cortex (Loeser and Treede, 2008; Morton et al., 2016). The reflex response is thought to be modulated by midbrain and thalamus (Morton et al., 2016), while part of the sensory–discriminative features of the pain processing entails the secondary somatosensory (S2) cortex, with the posterior insula (lateral network) (Ploner et al., 2002; Lockwood et al., 2013).

**FIGURE 1:**
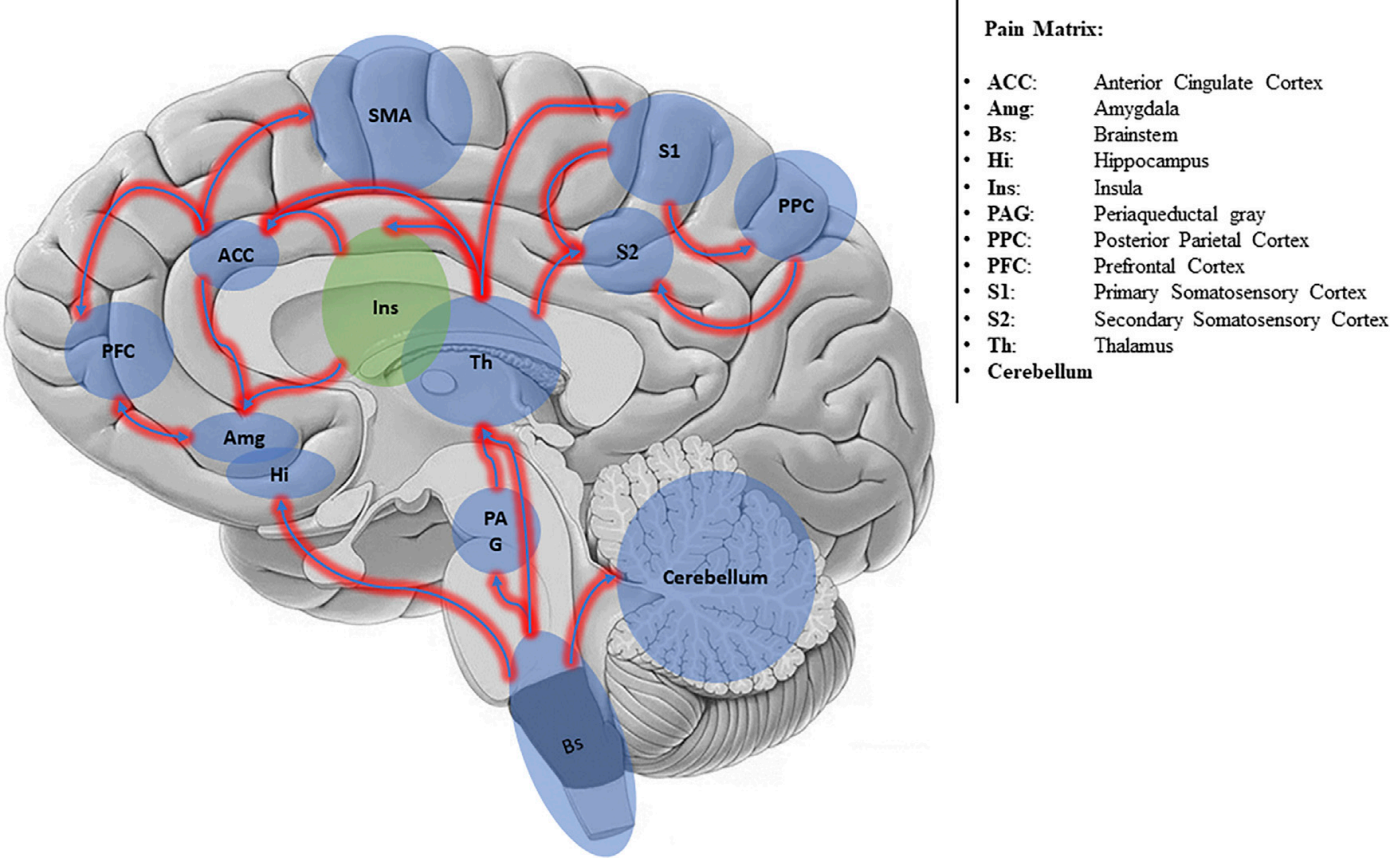
Simplified scheme of the Pain Matrix.

The conscious experience of pain requires a more complex network, generally called *Pain Matrix* (Iannetti and Mouraux, 2010; Salomons et al., 2016) (Figure 1).

The Pain Matrix involves two main subsystems: the Lateral Neuronal Network (LNN) and the Medial Network (MN). The LNN encompassing S2 cortex, lateral thalamus, and posterior insula (Mutschler et al., 2011) encodes the sensory discriminative information; the MN encompassing anterior cingulate cortex (ACC) and prefrontal cortex encodes affective-cognitive information (Medford and Critchley, 2010). Also, the cerebellum (Moulton et al., 2011) and motor areas (e.g., the striatum, cerebellum, and the supplementary motor area) (Barceló et al., 2012) are involved in pain perception and processing.

Several studies investigating the dynamics of activation (connectivity) of the pain matrix have shown that nociceptive input is first processed in the posterior insula, wherein it is coded in terms of intensity and anatomical location, and then transmitted to the anterior insula, where the emotional reaction to pain is elaborated (Ploner et al., 2002; Tracey, 2008; Frot et al., 2014).

For the "mind-body" theory, the pain experience necessitates of a body and a mental component (Duncan, 2000). The first encompasses the phenomena leading to perception and response, such as pain pathway and central processing, while the second encompasses perception and interpretation of pain, including the cognitive and affective components (Sarno, 2001). The mind-body approach shows the impossibility to separate mind and body in the pain experience, then the importance of self-report. Nevertheless, the IASP stated that “Verbal description is the only one of several behaviors to express pain; inability to communicate does not negate the possibility that a human or a nonhuman animal experiences pain” (IASP, 2020).

In non-communicative patients, the most relevant aspects in response to pain are physiologic (i.e., modification in the vital parameters such as heart rate and respiration) and behavioral (i.e., modification in the facial expression, motor and visual response).

To assess pain in non-communicative patients several behavioral scales were developed, with each of them oriented to assess a specific typology of patients. As an example, the Behavioral Pain Scale (Payen et al., 2001) which is commonly used in trauma or post-operative care unit to assess pain in critically sedated and mechanically ventilated patients; the Faces, Legs Cry and Consolability scale (FLACC) (Merkel et al., 2002; Malviya et al., 2006), which was developed for the pediatric population to measure pain severity; or the Pain Assessment in Advanced Dementia scale (PAINAD) (Warden et al., 2003), developed for patients affected by dementia.

Disorders of Consciousness (DOC) represent a spectrum of pathologies affecting the capacity of patients to interact with the external world. It can be either due to a traumatic or a non-traumatic cause and sometime to a combination of both (Giacino et al., 2018).

Among the different definition of consciousness, the most accepted viewpoint refers to the brain's ability to form cognition of the world, by the perception of self and the environment. A requisite for conscious behaviors is the presence of adequate arousal (i.e., wakefulness) and awareness of content (i.e., sensory, cognitive, and affective experience) (Giacino et al., 2018). The first is referred to the level of consciousness and the second to the content of consciousness (Xie et al., 2017; Giacino et al., 2018).

The two possible conditions following the acquired brain injury (i.e. a terrible event disrupting the arousal and awareness systems, mediated respectively by the brainstem and cortex) are either the Vegetative State/Unresponsive Wakefulness Syndrome (UWS/VS) or the Minimally Conscious State (MCS) (Giacino and Kalmar, 2005; Laureys et al., 2010; Giacino et al., 2018). The first is characterized by spontaneous opening of the eyes and no sign of consciousness, with only residual reflexive responses to external stimuli; the second by minimal but discernible signs of non-reflex behaviors (i.e., response to visual, auditory, tactile, or noxious stimuli) which occur in a reproducible even if inconsistent manner (Giacino et al., 2002; Schnakers et al., 2009).

The clinical assessment is based on clinical consensus and behavioral scales such as the Coma Recovery Scale (Giacino et al., 2004; Seel et al., 2010).

For the assessment of pain in patients with DOC a specific scale, the Nociception Coma Scale (Schnakers et al., 2010; Riganello et al., 2014) has been developed. It is based on the observation of the motor response (non/flaccid, abnormal posturing, flexion withdrawal, and localization to noxious stimulation), verbal response (non-verbalization, groaning, vocalization, and intelligible verbalization), visual response (none, startle, eyes movement and fixation) and facial expression (non-oral reflexive/startle response, grimace and cry), following a noxious stimulation (i.e., pressure on the fingernail bed using an algometer). Each subscale ranges from 0 (no response) to 3 (appropriate response), for a total score ranging from 0 to 12. A revised version, characterized by the absence of the visual subscale, was developed by Chatelle et al. (2016), but the two versions maintain the same clinimetric properties (Vink et al., 2017). Higher values for these scales indicate a more complex response to the noxious stimulus and content of consciousness.

A study by Sattin et al. (2018) reported lower pain pressure thresholds in DOC patients compared to healthy participants suggesting further investigations. Hyperestesia, hypoesthesia and anesthesia, conditions frequently present after acquired brain injury, may in fact alter responses to pain stimuli. Formisano and colleagues (Formisano et al., 2020) proposed for the evaluation of the response to painful stimuli by NCS and NCS-R, different and personalized stimuli (e.g., hand opening, upper limb abduction, head mobilization), because altered pain pathway may affect the searched responses by standard pressure on the fingernail bed.

### Pain and Consciousness in Disorders of Consciousness Patients

The treatments and management of pain is a challenging issue in patients with DOC. The condition of suffering in DOC patients is a very controversial question. Generally, caregivers and relatives believe in the possibility that VS/UWS patients might feel pain, influencing end-of-life decisions. However, there is not a unanimous consensus about whether non-responsive patients might have a sufferance condition or might feel pain (Demertzi et al., 2013; Demertzi, 2018), implying increasing ethical questions (Riganello et al., 2016).

Neuroimaging studies have shown different processing of pain between UWS/VS and MCS patients (Boly et al., 2008; Chatelle and Thibaut, 2014; Garcia-Larrea and Bastuji, 2018). In a seminal Oxigen 15 (O-H_2_O) PET study, pain induced activation of the midbrain, contralateral thalamus, and primary somatosensory cortex in UWS/VS patients (Laureys et al., 2002). Kassubek and colleagues, using the same PET technique in DOC patients, found the activation of the secondary somatosensory cortex, in the cingulate cortex contralateral to the stimulus, and the posterior insula ipsilateral to the stimulus (Kassubek et al., 2003). These findings suggest that DOC patients might have a residual perception and partial sensory-discriminative pain processing. However, the activation of the pain network resulted incomplete, with the primary somatosensory cortex functionally disconnected from the secondary somatosensory, bilateral posterior parietal, premotor, polysensory superior temporal, and prefrontal cortices (Laureys et al., 2004). The isolation of primary cortical activation from higher-order associative cortical activity suggests a non-integrated pain processing with a consequent less conscious experience (Boly et al., 2008).

Compared to the UWS/VS patients, MCS patients present higher metabolism in associative areas, principally in the precuneus/posterior cingulate cortex (Laureys et al., 2005), and a restoration of the correlation between these areas and the thalamus (Laureys et al., 2000). Boly and colleagues found similar brain area activation to noxious stimuli in MCS patients compared to controls (Boly et al., 2008) (i.e., thalamus, the primary somatosensory cortex, the secondary somatosensory cortex or insula, the posterior cingulate cortex/precuneus, and the anterior cingulate area). Another fMRI study performed by Markl and colleagues (Markl et al., 2013), demonstrated the significant activation of the sensory and affective components of the pain matrix in patients clinically diagnosed as UWS/VS, suggesting the possibility of a painful experience in some of these patients.

The neuroimaging, although it is a powerful tool of investigation, remains a complicated, expensive, time-consuming approach and of difficult use in the routine of clinical practice. In this frame, the behavioral pain assessment is still widely recognized as the most accessible and easiest approach. However, the risk of misdiagnosis remains high, considering that patients with DOC might not show any overt response to painful stimulation even if perceived (Schnakers et al., 2012; Chatelle and Thibaut, 2014; Calabrò et al., 2017; Cortese et al., 2020).

Different approaches of investigation based on Heart Variability Analysis (HRV), Galvanik Skin Response (GSR), or Laser Evocated Potential (LEP) have shown the possibility to observe pain processing in UWS/VS patients (de Tommaso et al., 2015; Riganello et al., 2018a; Cortese et al., 2020).

HRV is the fluctuation in the time intervals between adjacent heartbeats (interbeat interval - IBI) and represents the output of a complex brain-heart two-way interaction system (Riganello et al., 2012). The Central Autonomic Network (CAN), an integrative model where neural structures and heart function are involved and functionally linked in the affective, cognitive and autonomic regulation, describes this interaction (Benarroch, 2007; Thayer and Lane, 2009; Riganello, 2016). The principal neural structure of the CAN cover the brainstem (periaqueductal gray matter, nucleus ambiguous, and ventromedial medulla), limbic structures (amygdala and hypothalamus), prefrontal cortex (anterior cingulate, insula, orbitofrontal, and ventromedial cortex) and cerebellum (Benarroch, 2006, 2007; Lane et al., 2009; Thayer and Lane, 2009).

To describe the sympathovagal modulation, the HRV is generally analyzed in the time and frequency domains (Berntson et al., 1997). However, the physiological phenomena that characterize the biological events are dynamic and complex (Billman, 2011). For this reason, the non-linear analysis represents a useful approach to understand the brain-heart two-way interaction (Riganello, 2016). The HRV entropy quantifies the unpredictability and complexity of the IBI series. Higher and lower entropy indicate respectively higher or lower unpredictable IBI sequence, and correspondingly a higher or lower Heart-Brain two-way interaction (Riganello et al., 2018b). In a study based on noxious and non-noxious stimuli, lower HRV entropy was observed in UWS/VS compared to MCS patients and lower in MCS patients compared to healthy controls (Riganello et al., 2018a). Cortese and colleagues, through the GSR and HRV entropy measures, observed a trace conditioning of the nociceptive stimulus (i.e., a conditioning protocol where the Conditioned Stimulus - pain - is presented, terminated, and followed after some intervening period by the Unconditioned Stimulus - Music -) in patients diagnosed as UWS/VS and without any oriented or reflex behavioral response to the nociceptive stimulation (Cortese et al., 2020). The trace conditioning is considered an appropriate method to assess consciousness's presence without a verbal report (Bekinschtein et al., 2009). The GSR is an indicator of psychological or physiological arousal, measured by the skin conductance that is controlled by the sweat glands, that are controlled by the sympathetic nervous system (Critchley et al., 2000; Cernat et al., 2017). The GSR signal, used to observe the presence of the trace conditioning, was observed only in patients with UWS/VS who changed the level of consciousness within thirty days from the first assessment (suggesting the possibility in these patients to perceive and learn the pain stimulus). Moreover, the HRV entropy was higher in these patients compared to those that remain with the diagnosis of UWS/VS (Cortese et al., 2020).

In two different LEP studies, authors found that brain-injured UWS/VS patients might process the painful stimuli (de Tommaso et al., 2013; de Tommaso et al., 2015). In a subsequent study by Naro et al. (2016) on MCS and UWS/VS patients, authors reported the modulation of the γ-band oscillation power induced by nociceptive repetitive laser stimulations and its correlation with the NCS-R. The results showed a strong positive correlation between γ-band oscillation power and NCS-R in all MCS and some of the UWS/VS patients, suggesting that, also in the presence of a lower NCS-R total score, the UWS/VS patients may have had a covert pain’s experience. In a successive study, Calabrò and colleagues found γ-oscillations within the limbic system related to pain perception in some of the screened UWS/VS patients, evidencing that they might have perceived the affective component of pain (Calabrò et al., 2017).

### Pain in Disorders of Consciousness and Treatment

The above-cited results put in evidence two relevant points: firstly, the assessment of nociceptive stimulation as mean to detect possible content of consciousness in patients diagnosed as UWS/VS; secondly, also if not capable of exhibiting oriented behavior to the painful stimuli, UWS/VS patients might perceive pain. In a recent work, Cortese and colleagues (Cortese et al., 2020) showed that the increase of the score in the NCS anticipates the increase of the score in the CRS-R. This finding highlights the importance of pain assessment in these patients, and how the behavioral response to pain could precede other responsive behavioral aspects. However, the oriented behavioral response to the nociceptive stimuli could be covered by a necessary pharmacotherapy for the treatments of the suspected pain condition (Pistoia et al., 2015).

Pain could be present in the acute phase and in the successive period of intensive rehabilitation (Schnakers et al., 2012; Schnakers and Zasler, 2015). The cause of pain might arise from multiple factors such as skin lesions, surgical wounds, neuropathic pain, or injury of various types (i.e., abdominal, chest, fractures) as well as nursing-maneuvers with devices used during the hospitalization period (i.e., percutaneous endoscopic gastrostomy, nasogastric tube, bladder catheter replacement, venous and arterial blood sampling) (Ivanhoe and Hartman, 2004; Crooks et al., 2007; Baron, 2009; Popernack et al., 2015; Bexkens et al., 2017). In the rehabilitation as well as in the chronic phase, pain can arise from peripheral nerve lesions, central pain, diffuse spasticity, joint limitations, bedsores, paraosteoarthropathy, constipation, post-traumatic headache (Olver et al., 1996; Khan et al., 2003; Sherman et al., 2006; Hoffman et al., 2007; Ofek and Defrin, 2007; Baron, 2009; Gironda et al., 2009).

The Central Nervous System damage might be the cause of chronic pain (e.g., thalamic pain following a traumatic brain injury with diffuse axonal injury (Munivenkatappa and Agrawal, 2016; Irvine and Clark, 2018)). These conditions may lead to changes in the central nervous system pain processing and to a Complex Regional Pain Syndrome (CPRS), a neuropathic pain disorder characterized by distinct clinical features including allodynia, hyperalgesia, sudomotor and vasomotor abnormalities, and trophic changes (Schnakers et al., 2012; Guthmiller and Varacallo, 2020). Mechanism underlying CPRS is multifactorial, involving abnormal neuronal transmission, autonomic dysregulation, and central sensitization. The proinflammatory and immunological response increase production of interleukins, bradykinin, substance P, and osteoprotegerin, with consequent peripheral sensitization, alteration of the sympathetic nervous system and increasing expression of adrenergic receptors on nociceptive fibers (Guthmiller and Varacallo, 2020).

The presence of painful symptoms might interfere with the rehabilitation processes limiting and/or delaying its effect. It is crucial to intervene with appropriate early measure to prevent the appearance of secondary damage associated with pain and functional limitation such as bedsore or muscle-tendon retraction (Schnakers and Zasler, 2015).

In DOC patients, there is no general agreement on pharmacological pain treatment (Bartolo et al., 2016). Generally, it should be administered in the presence of behavioral signs of pain. The accurate pharmacotherapy dosage is crucial to avoid interferences with the assessment and treatment plan for the recovery of consciousness. Ineffective control of pain could affect or inhibit the emergence of intentional behavioral responses, while over-treatment could limit cognitive recovery and attention (Fins et al., 2008; Bartolo et al., 2016).

Brain lesions in these patients are extensive, affecting the nervous system at the cortical, subcortical, intracortical and spinal level. It is essential to provide basic care, managing the insurgence of the secondary medical complication that could increase the risk of further disability (Sazbon and Groswasser, 1990; Sazbon and Groswasser, 1991; Seel et al., 2013) and complicate their treatment and pain management.

Most of these patients are characterized by spasticity. The spasticity, due to a lesion of the pyramidal tract, is defined as “a motor disorder, characterized by a velocity-dependent increase in tonic stretch reflexes (muscle tone) with exaggerated tendon jerks, resulting from hyper-excitability of the stretch reflex as one component of the upper motor neuron syndrome.” (Lance, 1980). It is present in the 89% of DOC patients (Thibaut et al., 2015) and associated with pain and other symptoms such as increased hypertonia and altered sensorimotor control and muscle spasms (Burke et al., 2013). Infiltration of botulinum is advised in case of focal spasticity and to treat severe or worsening cases (Childers et al., 2004; Verplancke et al., 2005). In the case of dystonia and diffuse spasticity, an improvement in their management was observed by the intrathecal baclofen (Pistoia et al., 2015). The improvement of the level of consciousness in DOC was associated to the use of the intrathecal baclofen (Margetis et al., 2014; Pistoia et al., 2015), due probably to the reduced overload of the dysfunctional sensory stimuli reaching the brain or to the stabilization of the circadian rhythms (Margetis et al., 2014).

The symptomatic pain treatment follows the criteria of proportionality and graduality, assessing the interaction with the current therapy (Bartolo et al., 2016). The therapies approaches are generally based on aspirin, paracetamol, nonsteroidal anti-inflammatory drugs, opioid and γ-aminobutyric acid (GABA)-ergic agents (Mura et al., 2013; Bartolo et al., 2016). Aspirin, paracetamol, and nonsteroidal anti-inflammatory drugs should be administered in case of presumed mild pain (Schnakers and Zasler, 2007).

In case of suspected moderate pain and neuropathic pain, it is suggested a high-dose of aspirin or paracetamol, oral NSAIDs, GABAergic agents (Czuczwar and Patsalos, 2001; Enna and McCarson, 2006; Schnakers and Zasler, 2015; Bartolo et al., 2016). GABAergic agents are also indicated in case of psychomotor agitation or opposition to mobilization associable to pain. GABA is widely distributed throughout the neuraxis playing a central role in mediating or modulating most central nervous system functions. GABA_A_ and GABA_B_ receptors and GABAergic neurons are present in spinal cord and brain areas associated with the mediation and perception of pain (Enna and McCarson, 2006). Behavioral and physiological responses to pain are regulated by GABAergic projections from the ventral tegmental area and substantia nigra to the ventrolateral periaqueductal gray and dorsal medullary raphe nucleus (Kirouac et al., 2004). Both inhibitors of GABA uptake and metabolism and GABA receptor agonists display significant antinociceptive activity in animal models of acute, inflammatory, and neuropathic pain (Malan et al., 2002; Sa et al., 2004). Further, the antinociceptive response was observed to be induced by the activation of GABA_A_ receptors in the parafasciculus thalami (Reyes-Vazquez et al., 1986). The pharmacotherapy based on GABAergic agents may be accompanied by adverse effects such as drowsiness, fatigue, depression or constriction of the visual field (Czuczwar and Patsalos, 2001).

In the case of presumed severe pain, it is advised to consider the use of parenteral opioids, mixed agonists/antagonists, partial agonist opioids, antidepressants, anticonvulsants, and atypical agents (Schnakers and Zasler, 2015; Bartolo et al., 2016; Seal et al., 2018; Adams et al., 2020). The opioids act by binding proteins called opioid receptors that are widely distributed. Those involved in pain modulation are localized in the central and peripheral nervous system. These receptors also bind endorphins involved not only in pain modulation but also in other body functions such as reinforcement and reward mechanisms, mood and stress, mediated by deep structures of the brain (Russo and Nestler, 2013). The neural proliferation is also modulated by the opioid system (Sargeant et al., 2008) inducing, for example, neural degeneration (Atici et al., 2004; van Dijk et al., 2011) and apoptosis (Hu et al., 2002). Nevertheless, the use of opioids to treat analgesia may be accompanied by side effects, which will depend on the dose, such as somnolence, mental clouding, and respiratory depression (Rosenblum et al., 2008; Rogers et al., 2013) that might interfere with a correct diagnosis of the level of consciousness.

It is evident the current difficulty for pain treatment in patients with DOC, and the impossibility for the patient to refer on the pain perception makes the choice of the correct pharmacological approach a challenge.

At the light of these concerns, the guideline of the physicians should be based on the cost/benefit, intended as to follow the ethical principle of nonmaleficence/beneficence of the treatments.

## Conclusion

Pain is not only a perceptual phenomenon. The initial injury, cause of the pain, disrupts the body’s homeostatic systems which, in turn, produce stress. Pain involves a dynamic interaction among biological, psychological, and social factors. These components may modulate pain perception and disability (Duncan, 2000; Gatchel and Kishino, 2008).

The assessment and management of pain in patients with a DOC remain a challenge. The perception of pain in these patients arises rehabilitative problems with ethical issues extending beyond the boundaries of end-of-life decisions (Miller-Smith et al., 2019; Wolf-Meyer, 2020). To date, the correct assessment of DOC patients has a high rate of misdiagnosis (Bosco et al., 2010; van Erp et al., 2015), and the misinterpretation of the behavioral signs may lead to a non-fully appropriate rehabilitative approach.

The correct pain management and the capability to individuate the best pharmacological treatment can make the difference in detecting a behavioral response indicative of a change in the level of consciousness in DOC patients, and in planning a more effective rehabilitative approach.
